# Pharmacokinetic Models to Characterize the Absorption Phase and the Influence of a Proton Pump Inhibitor on the Overall Exposure of Dacomitinib

**DOI:** 10.3390/pharmaceutics12040330

**Published:** 2020-04-07

**Authors:** Ana Ruiz-Garcia, Weiwei Tan, Jerry Li, May Haughey, Joanna Masters, Jennifer Hibma, Swan Lin

**Affiliations:** 1 Metrum Research Group, San Diego, CA 92121, USA; anar@metrumrg.com; 2 Department of Pharmacometrics, Pfizer Inc, San Diego, CA 92121, USA; weiwei.tan@pfizer.com (W.T.); jerry.li@pfizer.com (J.L.); may.haughey@pfizer.com (M.H.); joanna.c.masters@pfizer.com (J.M.); jennifer.e.hibma@pfizer.com (J.H.)

**Keywords:** zero-order absorption, first-order absorption, combined zero- and first-order absorption, transit compartment absorption model

## Abstract

Introduction: Dacomitinib is an epidermal growth factor receptor (EGFR) inhibitor approved for the treatment of metastatic non-small cell lung cancer (NSCLC) in the first line in patients with EGFR activating mutations. Dacomitinib is taken orally once daily at 45 mg with or without food, until disease progression or unacceptable toxicity occurs. Oncology patients often can develop gastroesophageal reflux disease (GERD), which may require management with an acid-reducing agent. Proton pump inhibitors (PPIs), such as rabeprazole, inhibit sodium-potassium adenosine triphosphatase (H^+^/K^+^-ATPase) pumps that stimulate acid secretion in the stomach and have a prolonged pharmacodynamic effect that extends beyond 24 h post-administration. The aim of this work was to characterize the absorption of dacomitinib via modeling with a particular interest in quantifying the impact of rabeprazole on the pharmacokinetics (PK) of dacomitinib. Materials and Methods: The pooled dataset consisted of five clinical pharmacology healthy volunteer studies, which collected serial pharmacokinetic concentration-time profiles of dacomitinib. Non-linear mixed effects modeling was carried out to characterize dacomitinib pharmacokinetics in the presence and absence of the concomitant use of a PPI, rabeprazole. Several absorption models, some more empirical, and some more physiologically based, were tested: transit compartment, first-order absorption with and without lag time, and variations of combined zero- and first-order absorption kinetics models. Results: The presence of a PPI was a significant covariate affecting the extent (*F*) and rate (ka) of dacomitinib absorption, as previously reported in the dedicated clinical study. A transit compartment model was able to best describe the absorption phase of dacomitinib.

## 1. Introduction

Dacomitinib is currently approved for the first-line treatment of patients with metastatic non-small cell lung cancer (NSCLC) with epidermal growth factor receptor (EGFR) exon 19 deletion or exon 21 L858R substitution mutations [1]. Dacomitinib is a selective, adenosine triphosphate (ATP)-competitive, irreversible, small-molecule inhibitor of the HER (ErbB) family of receptor tyrosine kinases (RTKs), including the epidermal growth factor receptor (EGFR, HER1), the HER2 receptor (ErbB2), the HER4 receptor (ErbB4), and their oncogenic variants (e.g., EGFR with del exon 19 or L858R mutations) [2,3,4]. Dacomitinib has demonstrated the inhibition of HER1, HER2, and HER4 in biochemical kinase assays, the dose-dependent inhibition of HER1 and phosphorylation of HER2 RTK in tumor xenografts, and the inhibition of tumor growth or tumor regression in experimental models of cancer [5].

In clinical studies in healthy volunteers, following a single oral administration of dacomitinib at 45 mg under fasted conditions, the median time to reach the maximum observed plasma concentration (*T*_max_) of dacomitinib ranged from 8 to 10 h after dosing. Dacomitinib undergoes extensive extravascular distribution, with a geometric mean (percent coefficient of variation, CV%) apparent volume of distribution (*V*_z_/*F*) ranging from 2415 to 4005 L (19–33%). The in vitro binding of dacomitinib to human plasma proteins is approximately 98%. Following a single 45 mg oral dose of dacomitinib, the mean plasma half-life of dacomitinib ranged from 55 to 90 h, and the geometric mean apparent plasma clearance of dacomitinib was approximately 27 to 38 L/h [6,7,8,9,10].

In a Phase 1 crossover study, dacomitinib was administered as a single 45-mg oral dose under fasting conditions to subjects who had received seven daily doses of rabeprazole until steady-state was reached [6]. The median *T*_max_ of dacomitinib was approximately 12 h, longer than the *T*_max_ of 8 h seen with dacomitinib alone. Furthermore, exposure to dacomitinib, represented by area under the curve from 0 to 96 h (AUC_0–96_) and maximum concentration (*C*_max_), was reduced with coadministration with rabeprazole at steady state than when dacomitinib was given alone, with adjusted geometric mean ratios (GMR) for AUC_0–96_ and *C*_max_ of 60.8% and 49.5%, respectively.

The aim of this work was to characterize the PK of dacomitinib via modeling using healthy volunteer data, with a particular interest in quantifying the impact of rabeprazole, administered at a dose of 40 mg daily for 7 days prior to the administration of dacomitinib, on the absorption of dacomitinib.

## 2. Materials and Methods

The present population PK analysis was based on pooled data from 5 clinical studies in healthy volunteers. All healthy volunteers in the pooled dataset were male. A brief description of each study is provided in this section and in Table 1. For all PK assessments, the date and time of the clinic visit, and the dose, date, and time of the PK sample collection were captured on the case report form (CRF). The dates and times were used to derive the time that elapsed between the PK sample draw and last dose administered (hours post-dose).

Plasma samples were analyzed for the concentrations of dacomitinib at Intertek Pharmaceutical Services (San Diego, CA; formerly known as Alta Analytical Laboratory) using a validated, sensitive, and specific liquid chromatography atmospheric pressure ionization with tandem mass spectrometry (LC-API/MS/MS) method. The assay complied with Pfizer standard operating procedures (SOPs). The performance of the method during validation is documented at the clinical research organization (CRO)/Pfizer method validation report. Plasma specimens were stored at approximately −20 or −70 °C until analysis and assayed within the period of established stability data generated during validation. Calibration standard responses were linear over the range of 1.00 ng/mL to 200 ng/mL for dacomitinib using a weighted (l/concentration2) linear least squares regression. The lower limit of quantification (LLOQ) for dacomitinib was 1.00 ng/mL. Clinical specimens with plasma concentrations below the LLOQ were reported as below limit of quantification (BLQ). 

### 2.1. Pooled Analysis Dataset

The following studies were included in the pooled dataset and are summarized in Table 1. All studies were approved by an Independent Ethics Committee. All subjects provided written, informed consent prior to study entry. The studies were conducted in accordance with the Declaration of Helsinki and International Conference on Harmonization Good Clinical Practice Guidelines, in addition to meeting all local regulatory requirements. Adverse events (AEs) were monitored throughout the studies and recorded by the investigator, including severity (mild, moderate, or severe), and likely relationship to study treatment for all observed or volunteered AEs. Safety was also assessed by clinical laboratory tests, physical examination, measurement of vital signs (pulse rate and blood pressure), and electrocardiograms (ECGs).

#### 2.1.1. Study 1015

Study 1015 was an open-label, randomized, single-dose, 2-sequence, and 3-period crossover Phase 1 study to investigate the effect of food and the effect of increased gastric pH achieved by treatment with a proton pump inhibitor (PPI) on the PK behavior of dacomitinib in healthy adult subjects [6]. Subjects received a single 45-mg dose of dacomitinib under 3 different conditions or treatments (antacid treatment using rabeprazole, fasted, and fed), with treatments in Period 2 and Period 3 (fed/fasted) assigned in random order with a washout period of at least 16 days between treatments. PK samples were collected at specified times over 264 h post-dose in each period to determine plasma concentrations of dacomitinib.

The pooled data for this population PK analysis include all dacomitinib PK data from subjects who received a single 45-mg dose of dacomitinib with or without PPI under fasted condition and did not include the PK collections under fed conditions.

#### 2.1.2. Study 1021

Study 1021 was an open-label, single fixed sequence, 2 period Phase 1 study in healthy subjects who were CYP2D6 extensive metabolizers [7]. During Period 1, subjects received a single 45-mg dose of dacomitinib. During Period 2, subjects received a single 30-mg dose of paroxetine (a CYP2D6 inhibitor) once daily (QD) for 3 days (Days 1 to 3). On Day 4, subjects were co-administered a 45-mg single dose of dacomitinib plus a single 30-mg dose of paroxetine. Single 30-mg doses of paroxetine were administered QD for the next 6 days (Days 5 to 10). There was a washout period of at least 21 days between dose administration in Periods 1 and 2. Subjects were genotyped for CYP2D6 polymorphism. PK samples were collected at specified time points over 240 h post-dose in each period to determine the plasma concentrations of dacomitinib.

The pooled data for this population PK analysis included all dacomitinib PK data from subjects received a single 45-mg dose of dacomitinib without paroxetine under fasted conditions and did not include the PK collections when dacomitinib was given in combination with paroxetine.

#### 2.1.3. Study 1022

Study 1022 was a single-center, randomized, single-dose crossover study to determine the relative bioavailability of the proposed commercializable 45-mg dacomitinib tablet compared to three 15-mg clinical tablets in the fasted state in healthy subjects [8]. A total of 32 healthy men were enrolled and received treatment. PK samples were collected at specified time points over 264 h post-dose in each period to determine plasma concentrations of dacomitinib.

The pooled data for this population PK analysis included all dacomitinib PK data from all subjects who received a single 45-mg dose of dacomitinib in this study.

#### 2.1.4. Study 1039

Study 1039 was a single-center, randomized, single-dose, 2-treatment crossover study to investigate the effect of dacomitinib co-administration on dextromethorphan exposure, a CYP2D6 probe drug, in healthy adult subjects [9]. Subjects received 2 treatments (Treatment A was single 30-mg oral dose of dextromethorphan; and Treatment B was 45 mg single oral dose of dacomitinib followed 4 h later by a single 30-mg oral dose of dextromethorphan) in random order following an 8-h fast with a washout period of at least 14 days between treatments. Subjects were genotyped for CYP2D6 polymorphism. Serial PK samples were collected at specified times over 144 h (dacomitinib) and 48 h (dextromethorphan) post-dose in each period to determine the plasma concentrations of dextromethorphan, dextrorphan, and dacomitinib. When dacomitinib was co-administered with dextromethorphan, the exposure (AUC and *C*_max_) of dextromethorphan was markedly increased (855.4% and 873.5%, respectively). These results suggest that dacomitinib may increase the exposure of other drugs primarily metabolized by CYP2D6.

The pooled data for this population PK analysis include all dacomitinib PK data from all subjects that received a single 45-mg dose of dacomitinib in this study. It should be noted that dextromethorphan did not impact dacomitinib PK; therefore, all dacomitinib PK data from this study was included.

#### 2.1.5. Study 1051

Study 1051 was a single-center, open-label, 1-period, single-dose study to determine the PK of dacomitinib, in healthy male adult Chinese subjects following a single oral dose of 45 mg dacomitinib administered under fasted conditions [10]. Serial PK samples were collected at specified time points over 11 days to determine the plasma concentrations of dacomitinib. A total of 14 healthy Asian subjects were enrolled, received treatment, and were evaluated for PK.

The pooled data for this population PK analysis include all dacomitinib PK data from all subjects that received a single 45-mg dose of dacomitinib in this study.

### 2.2. Modeling Software and Analysis

All modeling was performed using the NONMEM version 7.4.3 software (ICON Development Solutions, Ellicott City, MD, USA). The stepwise covariate model building procedure (SCM) was executed using Perl-speaks-NONMEM (PsN), version 4.9.0 [11]. For data manipulation, visual predictive checks (VPCs), post-processing, and plotting, R version 3.5.1 (R Foundation for Statistical Computing, Vienna, Austria) was used [12]. The NONMEM population PK dataset included patient identification, dosing information, the time of sample collection, serum concentrations, and other relevant information (e.g., demographics, laboratory test values).

During model building, the goodness of fit (GOF) of different models to the data was evaluated using the following criteria: (1) change in the objective function value (OFV), (2) visual inspection of scatter plots, (3) precision of the parameter estimates, and (4) decreases in both inter-individual variability (IIV) and residual variability. These criteria were used only when the minimization step was successful and standard errors of parameter estimates were obtained using the covariance step. The difference in the OFV between 2 hierarchical models has an approximate *χ*^2^ (chi-square) probability distribution with the number of degrees of freedom (df) equal to the difference in the number of parameters between the models. Based on the *χ*^2^ distribution with df = 1, a change in OFV of 10.83 corresponds to a significance level (*α*) of 0.001.

The stochastic approximation expectation-maximization (SAEM) estimation method with interaction available in NONMEM was used in the analysis. This method leads to population parameters converging towards the maximum of the exact likelihood. The OFV that is displayed during SAEM analysis is not valid for assessing minimization or for hypothesis testing. It is highly stochastic, and does not represent a marginal likelihood that is integrated over all possible empirical Bayes prediction of the interindividual random effect (ETA, *η*), but rather, is the likelihood for a given set of *η*s. After the stochastic portion was completed, a suitable objective function for hypothesis testing and standard errors was obtained by importance sampling method (IMP) at the final population parameter values [13]. During model building, the GOF of different models to the data was evaluated.

The stability of the models throughout the model development process was closely evaluated. Inspection of the covariance matrix of the estimates at every stage of model development was performed to verify that extreme pairwise correlations of the parameters were not encountered and avoid ill conditioning. Additionally, it was ensured that the condition number of the covariance matrix of the parameter estimates (i.e., the ratio of the largest to smallest eigenvalues, obtained from the PRINT = E option on the covariance block) was less than 1000 [14].

The IIV in the PK parameters was modeled using multiplicative exponential random effects of the form:

(1) $$\theta_{i}=\theta \cdot e^{\eta_{i}}$$

where *θ* (THETA) is the typical individual (population mean) value of the parameter and *η_i_* denotes the interindividual random effect accounting for the i^th^ individual’s deviation from the typical value having zero mean and variance *ω*^2^. The approximate CV% was reported as:

(2) $$\%CV=\sqrt{\omega^{2}}\cdot 100\%$$

The multivariate vector of interindividual random effects (across parameters within each individual) has variance-covariance matrix *Ω* (OMEGA). The diagonal *Ω* matrix was applied first and other (unstructured) *Ω* block structures were also explored by examining the potential correlations among all the empirical Bayes estimates (“post-hoc”) of the interindividual random effects (*η*s) with the focus on the correlation of the central compartment parameters (e.g., between CL and V). The *Ω* block was built only if the correlation was observed.

Residual variability (*ε*) was modeled additively based on log-transformed observation data using thetarized variance-covariance matrix of the intraindividual random effects (*σ*, SIGMA):

(3) $$\ln\left(Y_{ij}\right)=\ln\left(F_{ij}\right)+W\cdot \varepsilon_{ij}$$

where ln(*Y_ij_*) denotes the observed concentration for the i^th^ patient at time *t*_j_ on logarithm scale, the ln(*F_ij_*) denotes the corresponding model-predicted concentration on logarithm scale, and *ε_ij_* denotes the intraindividual random effect, assumed to have a mean of zero and variance *σ*^2^ of 1. W was the estimated variance of the residual variability that was one of the *θ*s to be estimated.

Adequacy of model fit was assessed through review of diagnostic plots. The result of this stage of model development was considered the final base model.

The only covariate considered in this analysis was the presence or absence of proton pump inhibitor, rabeprazole, on the absorption parameters. PPI was tested for significance in a stepwise manner with statistical criteria of *α* = 0.05 for the forward inclusion step, which corresponds to an OFV change of 3.84 based on a *χ*^2^ distribution with df = 1. The full model was then subjected to a backward elimination step with a statistical criterion of *α* = 0.001, which corresponds to an OFV change of 10.83 based on a *χ*^2^ distribution with df = 1. In order to obtain the most parsimonious and stable final model, the candidate covariate model resulting from the backward elimination step in SCM was subjected to a separate NONMEM run with a $COV step executed to examine any sign of model over parameterization and poorly estimated parameters.

Model adequacy, possible lack of fit, and the violation of assumptions were assessed at all stages of model development. Diagnostic plots of observations (OBS) versus Monte-Carlo-generated population predictions (EPRED) and OBS versus individual predictions (IPRED) were evaluated for randomness around the line of unity. Evaluation was also performed on the longitudinal profiles of PK concentration to compare observations and predictions. The plots of conditional weighted residuals (CWRES), individual weighted residuals (IWRES), and normalized prediction distribution errors (NPDE) versus EPRED and time after dose were evaluated for randomness around the zero line. The distribution of *η*s was checked to ensure approximately normal distribution.

In addition, the plots of *η*s in the final model versus the presence or absence of PPI were compared to similar plots for the base model to demonstrate that the final model accounted for trends observed with the base model. The 95% CI around the parameter estimates were generated based on standard error (SE) generated from the NONMEM covariance step.

A comparison of the OFV statistics and parameter estimates for the base and final models was used to assess the degree of parsimony of the final model and to determine the statistical relevance of the covariate effects. A comparison of *ω*^2^ between the models was made to assess the reduction in parameter variability by the inclusion of covariate effects.

Both η-shrinkage (1-SD[*η*]/*w*) and *ε*-shrinkage (1-SD[IWRES]) were evaluated to assess the validity of using post-hoc individual parameter estimates for model diagnosis [15].

The performance of the final model was evaluated by simulating data using the parameter estimates from the final model (fixed and random effects) and conducting a VPC. Simulations were performed using the patients’ characteristics as well as the dosing and sampling history from the original dataset. From these simulations, concentration time data were summarized using median (50th), low (2.5th), and high (97.5th) percentiles. The concordance between individual observations and simulated values as well as the distribution of observed and simulated data were evaluated [16,17].

### 2.3. Absorption Models

The selected studies for this analysis were performed in healthy volunteer subjects under a well-controlled environment where no medications were allowed during the conduct of the trial, with the exception of rabeprazole, that could somehow interfere in the absorption or disposition of dacomitinib. Of note, rabeprazole is not known to impact the metabolism of dacomitinib; thus, the testing of the PPI effect on dacomitinib only occurred on absorption parameters.

Dacomitinib undergoes oxidative metabolism and glutathione conjugation. The oxidative metabolism of dacomitinib involves cytochrome P450 (CYP) 2D6 for the formation of O-desmethyl-dacomitinib and CYP3A for the formation of other minor oxidative metabolites [7,18]. The change in relative bioavailability (*F*) estimated in this study was the result of the relative change in absorption fraction, as the first pass metabolism should remain the same.

A two-compartment disposition model characterized well the disposition of dacomitinib and was implemented for this analysis [19]. Four different absorption models were tested:

*First-order absorption:* The disappearance of the drug from the gastrointestinal (GI) tract occurs by a first-order process characterized by an absorption rate constant, *k_a_*. This model was tested with and without the addition of one more parameter: lag time (*t*_lag_). *T*_lag_ often improves the model fit by shifting the time of dosing as if the drug were administered at a later time.

*Transit compartment:* This model helps with delayed absorption profiles describing drug absorption as a multiple step process represented by a chain of pre-systemic compartments:

(4) $$\frac{da_{n}}{dt}=k_{tr}\cdot a_{\left(n-1\right)}-k_{tr}\cdot a_{n}$$

where *da_n_*/*dt* is the rate of change of amount of drug on compartment *n* at time *t*, *a_n_* is the drug amount in the n^th^ compartment at time *t*, *k_tr_* is the transit rate constant, and n is the number of transit compartments.

Using Stirling approximation to *n*!, the number of compartments to include can be estimated, avoiding stepwise addition of one compartment at a time.

(5) $$a_{n}\left(t\right)=F\cdot \mathrm{Dose}\cdot \frac{{\left(k_{tr}\cdot t\right)}^{n}}{n!}\cdot e^{-k_{tr}\cdot t}$$

The approximation of Stirling to *n*!:

(6) $$n!\approx \sqrt{2\pi }\cdot n^{n+0.5}\cdot e^{-n}$$

The disappearance of drug from the absorption compartment (*dA_a_*/*dt*) will be:

(7) $$\frac{dA_{a}}{dt}=\mathrm{Dose}\cdot F\cdot k_{tr}\cdot \frac{{\left(k_{tr}\cdot t\right)}^{n}\cdot e^{-k_{tr}\cdot t}}{\sqrt{2\pi }\cdot n^{n+0.5}\cdot e^{-n}}-k_{a}\cdot A_{a}$$

This model estimates a mean time of transit (MTT) parameter, which represents the average time spent by dacomitinib traveling from the first transit compartment to the absorption compartment [20]. The number of transit compartments was estimated in the base structural model and subsequently fixed for the characterization of the effect of rabeprazole effect on dacomitinib absorption.

*Sequential independent zero-order and first-order model:* It is assumed that two different kinetic absorption processes take place. First, a fraction of the dose, F2, is absorbed by zero-order kinetics during a given period of time (D2). The remaining fraction of the dose (1 − F2) is absorbed by first-order kinetics, characterized by ka. These two processes happen sequentially, first the zero-order, followed by the first-order absorption [21]. The database should include two records with the full dose (AMT), one with RATE = −2 and CMT = 2 (zero-order absorption kinetics to the central compartment) and another one with RATE = 0 and CMT = 1 (first-order absorption kinetics from the gastrointestinal (GI) compartment to the central compartment).

*Sequential but linked zero- and first-order absorption:* A more mechanistic model assumes that the initial absorption process follows zero-order kinetics and is limited by the solubility of dacomitinib in the GI tract fluid [21]. The database format should be similar, as indicated with sequential independent zero- and first-order kinetics. Zero-order kinetics assumes the volume of gut fluid is constant. The duration of this zero-order absorption (D2) ends when the dacomitinib dose has completely dissolved and all of the drug is in solution. The rest of the dose in the GI tract (1 − F2) follows a first-order absorption process characterized by an absorption rate constant *k_a_*. By assuming a link between these two processes, the absorption constant, *k_a_*, does not need to be estimated but rather is derived from F2 and D2 as follows:

(8) $$\mathrm{Absorption}\;\mathrm{zero}\;\mathrm{rate}=\frac{F2\cdot \mathrm{Dose}}{D2}$$

Thus, the first order absorption rate constant will begin for all the remaining drug, which is no longer a saturated solution:

(9) $$\mathrm{Absorption}\;\mathrm{zero}\;\mathrm{rate}=\frac{F2\cdot \mathrm{Dose}}{D2}=k_{a}\cdot \left(1-F2\right)\cdot \mathrm{Dose}$$

(10) $$k_{a}=\frac{F2}{\left(1-F2\right)\cdot D2}$$

As these analyses estimated changes in relative bioavailability, the bioavailability parameter was fixed to 1. In presence of rabeprazole, changes to the relative bioavailability as well as to the parameters involved in the absorption process (D2, *k_a_*, and MTT) were screened via stepwise covariate modeling.

## 3. Results

**Figure 1:**
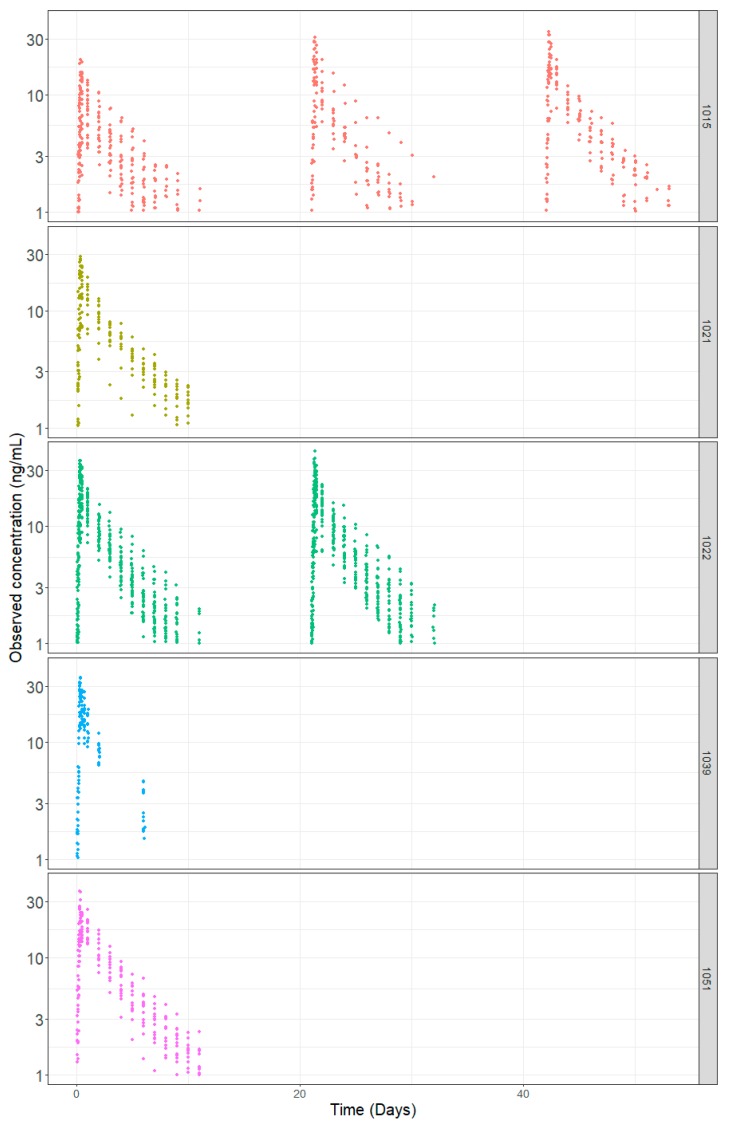
Dacomitinib Concentration-Time Profiles by Study.

Summary statistics for the different demographic factors: baseline body weight and age are shown in Table 2. The population comprised only male subjects with moderate variability in age and body weight. Figure 1 depicts the observed dacomitinib concentration time profiles using time after first dose by study.

**Figure 2:**
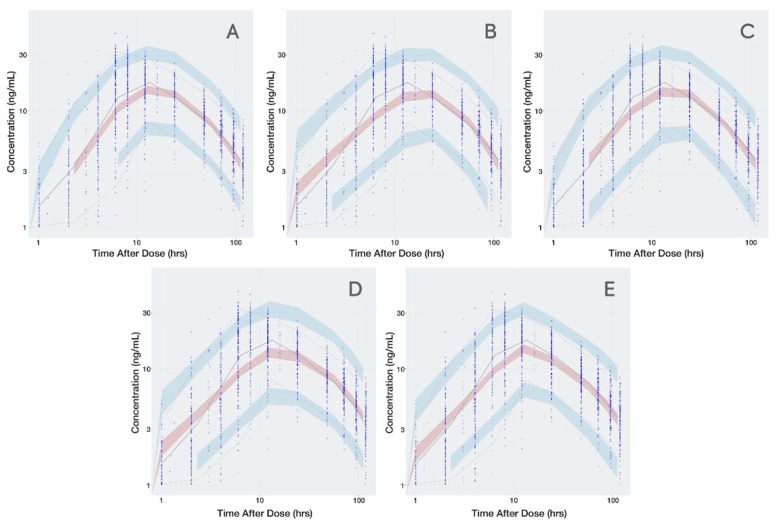
Visual Predictive Check for all Dacomitinib Base Models. (**A**): transit compartment model; (**B**): first-order absorption without lag time; (**C**): first-order absorption with lag time; (**D**): sequential but linked zero- and first-order absorption; (**E**): sequential independent zero- and first-order absorption. Time is presented in logarithmic scale to stretch the time around *C*_max_ and better appreciate how well the absorption phase is captured. Observed data are presented in blue circles with the 50th percentile of the observed data represented by the solid blue line and the 5th and 95th percentiles of the observed data represented by the dotted blue lines. Red shaded region represents the prediction interval for the 50th percentile. Blue shaded regions represent the prediction intervals at the 5th and 95th percentiles.

The transit compartment model presented a significantly lower OFV than the first-order or combined zero- and first-order absorption models (Supplementary Material, Table S1). The condition number for the transit compartment model did not suggest ill conditioning and the VPC (Figure 2) captured the *C*_max_ better relative to the other base structural models. Table 3 summarizes the base model parameters for all 4 models tested.

The differences in PK parameter estimates obtained with the different models were more pronounced for the apparent distribution and clearance parameters, where F was fixed to 1 (*CL*, *V*, *Q*, and *V*ss) than for the absorption parameters (*k_a_*, D2, and MTT), which are not associated to F. The estimated number of transit compartments for dacomitinib was 1.

Therefore, the transit model was carried forward for the characterization effect of rabeprazole on the dacomitinib absorption phase. The base model was then subjected to graphical examination (*η*s versus PPI) to investigate whether the estimated parameters captured the PPI effect.

**Figure 3:**
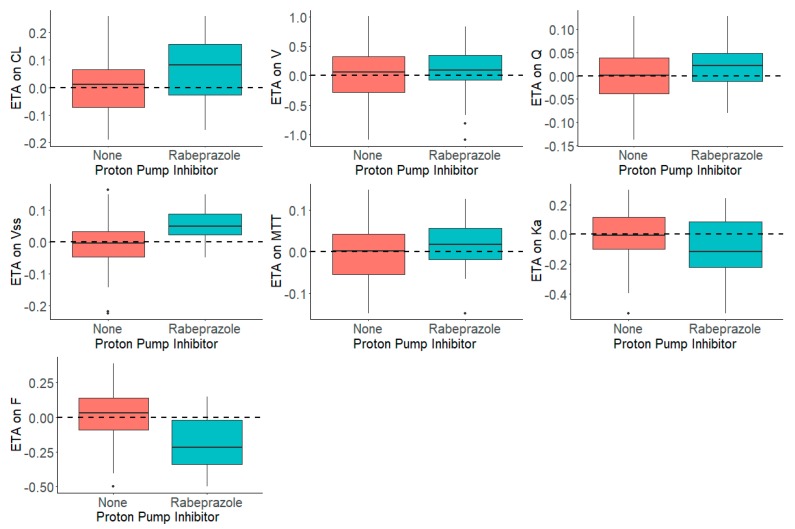
Distribution of *η*s (ETAs) by the Presence and Absence of Proton Pump Inhibitor, Rabeprazole, in the Dacomitinib Base Model.

**Figure 4:**
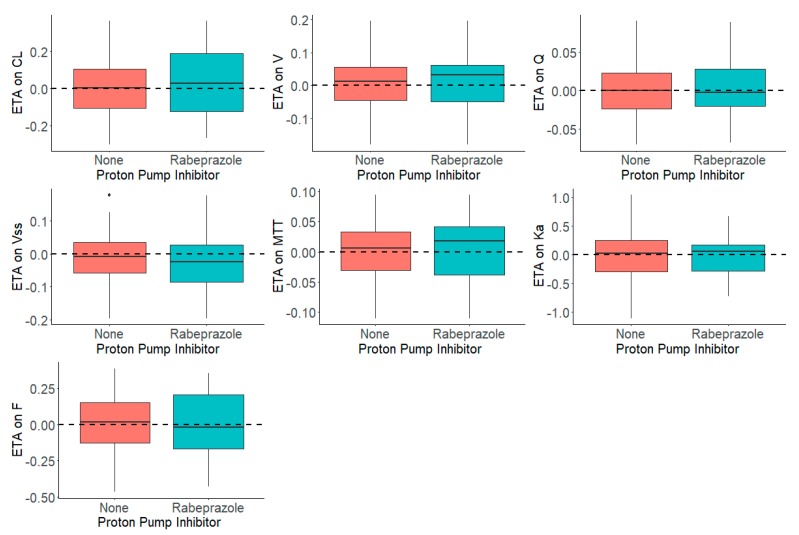
Distribution of *η*s (ETAs) by the Presence and Absence of Proton Pump Inhibitor, Rabeprazole, in the Dacomitinib Final Model.

Figure 3 shows the distribution of the *η*s in presence and absence of rabeprazole for each parameter. The presence and absence of rabeprazole was tested for significance in a stepwise manner with statistical criteria of *α* = 0.05 for forward inclusion Using SCM, the full model was reached in three forward selection steps and included the effects of rabeprazole on F and ka. After one backward elimination step (*α* = 0.001, removal of any of these covariates resulted in a more than 10.83 increase in OFV), the final model was achieved. None of the selected parameters identified in the full model were removed in the backward step. The final model, including the covariates, was further tested in NONMEM with a $COV step executed to examine any sign of model over parameterization and poorly estimated parameters. Figure 4 shows that the inclusion of rabeprazole on *F* and *k_a_* in the final model corrected for the previously observed trend in plots of *η*s on apparent CL and volume and *k_a_*. Table 4 tabulates the final parameter estimates for the transit compartment model with the addition of PPI as a covariate on *F* and *k_a_*.

**Figure 5:**
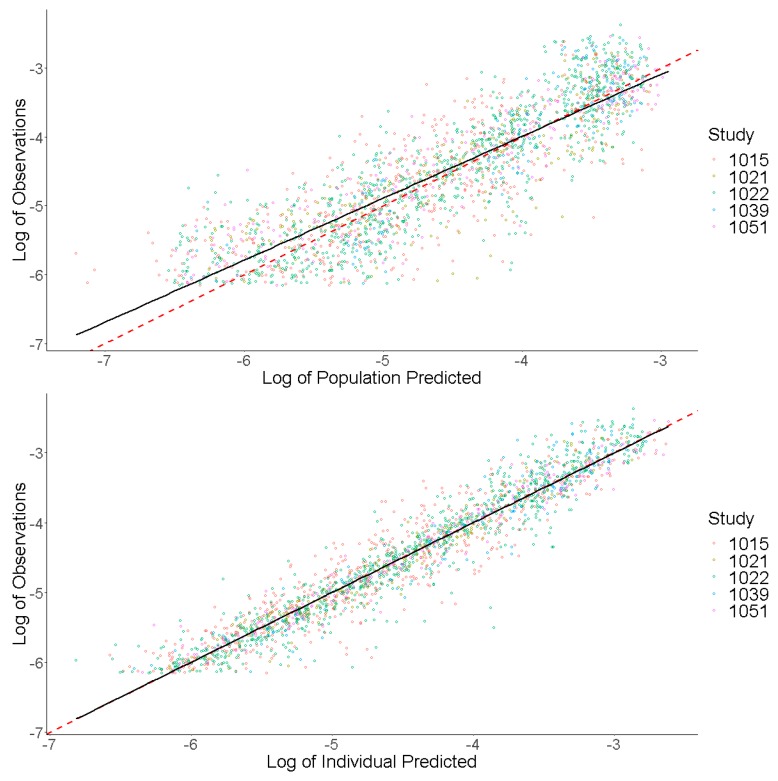
Prediction-Based Diagnostics for Dacomitinib Final Model. Observed concentrations (log transformed) are presented on the *y*-axis. Individual predicted concentrations (log transformed) are presented on the *x*-axis. The red dashed line represents a line of unity, and the black line represents a linear smooth line.

**Figure 6:**
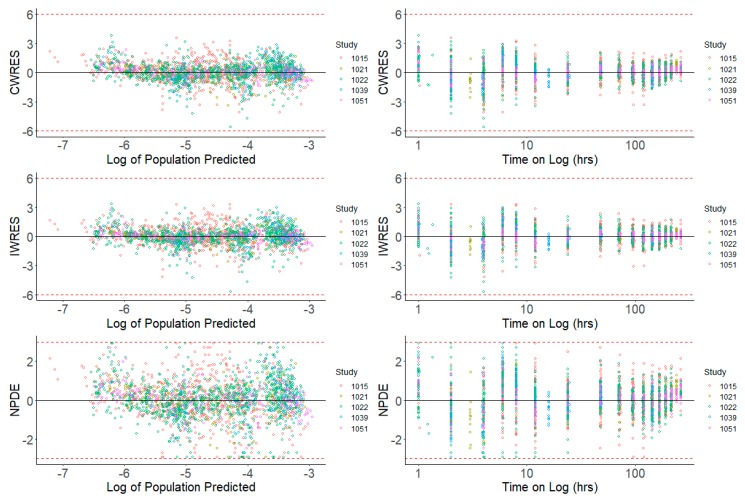
Residual-Based Diagnostics for Dacomitinib Final Model. Residual-based diagnostic plots of conditional weighted residuals (CWRES), individual weighted residuals (IWRES), and normalized prediction distribution errors (NPDE) versus population predicted concentrations (log transformed) on the left side, and time (on log scale) on the right side.

Prediction-based diagnostic plots (Figure 5) on the final model comparing OBS versus EPRED showed that the population prediction was a reasonable measure of central tendency of the data. Given that the *ε*-shrinkage was only 6.44% in the final model, the plot of OBS versus IPRED was informative and could be used to examine any model misspecification. The magnitude of the spread for OBS versus IPRED was small around the line of identity, indicating that the model predicted the observed concentrations well. Residual-based plots of IWRES, CWRES, and NPDE plotted against population predictions did not indicate any model misspecification of structural model or residual error model (Figure 6). In the plots of IWRES, CWRES, and NPDE versus time after dose, the majority of the data were evenly distributed across the *x*-axis, indicating no major deviation or trend over the entire observation time in the population (Figure 6).

**Figure 7:**
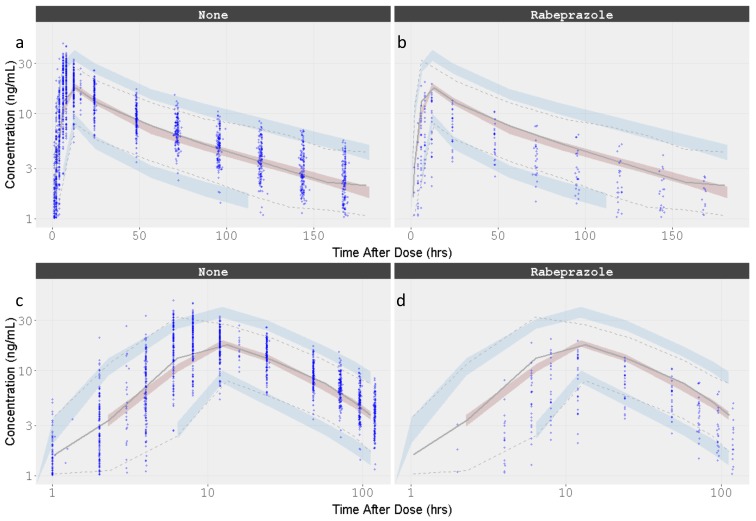
Visual Predictive Check for the Dacomitinib Final Model by Concomitant administration of Rabeprazole. Time is presented in linear and logarithmic scale to stretch the time around *C*_max_ and better appreciate how well the absorption phase is captured. (**a**) Dacomitinib without rabeprazole co-administation; (**b**) Dacomitinib with rabeprazole co-administation; (**c**) Dacomitinib without rabeprazole co-administation with time in the log scale; (**d**) Dacomitinib with rabeprazole co-administation with time in the log scale. Observed data are presented in blue circles with the 50th percentile of the observed data represented by the solid blue line and the 5th and 95th percentiles of the observed data represented by the dotted blue lines. Red shaded region represents the prediction interval for the 50th percentile. Blue shaded regions represent the prediction intervals at the 5th and 95th percentiles.

VPCs were performed for the final model, plotting the 5th, 50th, and 95th percentiles for observed data, and the 95% CIs around these percentiles for simulated data. The final model had good predictive performance, with the 5th, 50th, and 95th percentiles of the observed data lying within the 95% prediction intervals of the simulated 5th, 50th, and 95th percentiles. The results are displayed in Figure 7.

## 4. Discussion

Drug absorption is a complex process dependent on several variables, including pharmaceutical form (immediate release formulation, control release formulation, etc.), which determines the liberation of the drug from the formulation, physicochemical properties of the drug, and the physiological processes of the GI. Dissolution, solubility and permeability driven by the physicochemical properties of the active ingredient are the main factors affecting drug absorption, as reported by the Biopharmaceutical Classification System (BCS) [22,23,24]. Several physiological processes may affect drug absorption, including gut motility, pH, gastric emptying rate, and metabolism in the gut wall. The rate, extent, and length of time before the drug appears in the systemic circulation are often determined by a combination of these factors.

The use of physiology-based absorption models has been developed to account for physicochemical properties as well as many physiological processes. These mechanistic models require extensive prior knowledge not usually available, preventing the routine application of them in drug absorption estimation. The transit compartment model approximates to a physiological model as the presence of transit compartments describes the concentration-time profiles as a gradually increasing continuous function. Transit compartments describe a delay in absorption or a prolonged absorption phase as drug moves through a chain of identical compartments that are linked to the central compartment by a first-order absorption process explaining the delay in absorption in a smoother way than the lag time.

The parameter estimate D2, obtained with the base structural model for linked (9.78 h) and independent (10.4 h) combination kinetics, and the final MTT estimate (12 h) are all of similar magnitude, indicating that the absorption process takes place over at least 12 h, which is close to the observed *T*_max_ (see Figure 2 and Figure 7). However, the estimated lag time was 0.749 h, which is close to the first concentration after dosing (1 h), suggesting that this parameter may be biased by the collection time of the first sampling.

This dacomitinib population pharmacokinetic analysis was based on pooled data from five clinical trials in healthy volunteer studies under well-controlled conditions. In these studies, dacomitinib was administered as a single dose under fasting conditions. Study 1015 concluded that there was no effect of food on dacomitinib PK; as such, the prescribing labels recommend dosing dacomitinib with or without food. This pooled population PK analysis demonstrated that a 2-compartment PK model with one transit compartment and a mean transit time of 12 h followed by a first-order constant rate of absorption accurately described the concentration time course of dacomitinib. Rabeprazole was a statistically significant predictor of relative bioavailability and ka variability. When rabeprazole is concomitantly administered with dacomitinib, the relative bioavailability and absorption rate constant of dacomitinib decreased by 52% and 49%, respectively.

For class II compounds, such as dacomitinib, for which solubility is pH dependent (the water solubility of dacomitinib dramatically decreases as pH exceeds 4.5), the administration of acid-reducing drugs may affect bioavailability (refer to Supplementary Material, Figure S1, for dacomitinib chemical structure and pKa). This is of particular concern in cancer patients, as concomitant use of acid-reducing agents such as PPIs is common, and several other small-molecule tyrosine kinase inhibitors (TKIs) undergo changes in drug exposure with use of these agents. The effects of the coadministration of a TKI with an acid-reducing agent have been reported with erlotinib [25,26], nilotinib [27], gefitinib [28], bosutinib [29], lapatinib [30], neratinib [31], nilotinib [32], and pazopanib [33], decreasing AUC by 46%, 34%, 44%, 26%, 26%, 65%, 34%, and 40%, respectively. Therefore, with anticancer agents, there is concern for a risk of not achieving therapeutic plasma concentrations.

## 5. Conclusions

Cancer patients are heavily medicated and may develop GERD as a result of anti-cancer treatment and progressive disease. Thus, patients often require acid-reducing agents for gastroprotection and symptom management. Based on the conclusions from Study 1015 and the population PK modeling, PPIs are not recommended to be used with dacomitinib treatment. Unlike the prolonged effect of PPIs, local antacids have a mild and short-acting acid-reducing effect. Patients treated with dacomitinib may be able to use shorter-acting acid-reducing agents, such as H_2_-receptor antagonists and local antacids, and avoid treatment with PPIs.

## Figures and Tables

**Table 1:** Dacomitinib population PK pooled dataset—summary of studies.

Protocol No.	Study Design and Objective	Treatment Groups	No. of Subjects	Duration of Treatment	Study Start/Status
1015	Randomized, single-dose, 2-sequence, and 3-period crossover Phase 1 study To estimate the BA of a single 45-mg dose of dacomitinib under fed and fasted conditions. To estimate the BA of a single 45-mg dose of dacomitinib administered under antacid drug treatment relative to fasted conditions. To evaluate the safety and tolerability of the proposed formulation in healthy subjects.	Healthy volunteers (Route: oral; Dose Regimen: single 45-mg dose of dacomitinib) 3 conditions (A: antacid treatment, B: fasted, and C: fed)	Planned: 24 Randomized: 24	Single dose each treatment period; 12 Study Days each treatment period with at least a 16-day washout period between each dose.	25 Oct 2012/21 Jan 2013
1021	Open-label, single-fixed sequence, 2-period Phase 1 assessment in extensive CYP2D6 metabolizer subjects To estimate the effect of paroxetine, on the PK of a single 45-mg dose of dacomitinib. To assess the safety and tolerability of dacomitinib when given alone and when co-administered with paroxetine.	Healthy volunteers (Route: oral; Dose Regimen: Dacomitinib: 45-mg QD. Paroxetine: 30 mg QD) Schedule shown in “Duration of Treatment.”	Planned: 14 Randomized: 14	Period 1 (11 days): single 45-mg dose of dacomitinib on Day 1. Period 2 (14 days): single 30-mg doses of paroxetine QD for 3 days, then single 45-mg dose of dacomitinib plus a single 30-mg dose of paroxetine on Day 4. On Days 5 to 10, single 30-mg doses of paroxetine were administered QD. There was washout period of at least 21 days between periods.	28 Mar 2011/08 Jun 2011
1022	Randomized, single-dose, 2-sequence, 2-period crossover Phase 1 study To determine the relative BA of the proposed 45-mg dacomitinib tablet to 3 × the clinical 15-mg tablet. To assess the inter-subject variability in dacomitinib plasma PK of the proposed 45-mg dacomitinib tablet compared to 3 × the clinical 15-mg tablet in the fasted state. To evaluate the safety and tolerability of the proposed 45-mg dacomitinib tablet and the clinical formulation.	Two treatment periods: Treatment A: 3 × 15-mg single oral dose of, clinical tablets. Treatment B: 45 mg single oral dose of the proposed 45 mg dacomitinib tablet.	Planned: 32 Randomized: 32	Single dose each treatment period; 12 Study Days each treatment period with at least a 16-day washout period between each dose.	04 Apr 2011/20 May 2011
1039	Open-label, randomized, 2-period, 2-treatment, 2-sequence, cross-over, single-dose Phase 1 study. To estimate the effect of a single 45-mg dose of dacomitinib on the PK of a single 30-mg dose of dextromethorphan. To assess the PK of a single dose of dacomitinib and to assess safety and tolerability of dacomitinib and dextromethorphan.	Healthy volunteers (Route: oral; Dose Regimen: Treatment A: A single 30-mg dose of dextro-methorphan. Treatment B: A 45-mg dacomitinib, followed 4 h later by 30 mg of dextro-methorphan)	Planned: 14 Randomized: 14	Two treatment periods followed by a washout period of at least 14 days.	30 Oct 2009/17 Dec 2009
1051	Open-label, non-randomized, 1 period Phase 1 study to characterize the PK of dacomitinib. To characterize the PK of a single 45 mg oral dose of dacomitinib administered under fasted conditions to healthy Chinese volunteers. To evaluate the safety and tolerability of a single 45 mg oral dose of dacomitinib administered under fasted conditions to healthy Chinese volunteers.	Healthy Chinese volunteers (Route: oral; Dose Regimen: single 45-mg dose of dacomitinib)	Planned: 14 Randomized: 14	Single oral dose; total of 12 Study Days.	31 Jul 2014/04 Sep 2014

Abbreviations: BA = bioavailability; CYP = cytochrome P450; PK = pharmacokinetics; QD = once daily.

**Table 2:** Demographic Characteristics for the Pooled Dataset of Dacomitinib Studies.

Study	n	Age (Years) Mean (SD)	Age (Years) Median (Min–Max)	Body Weight (kg) Mean (SD)	Body Weight (kg) Median (Min–Max)
**1015**	24	36.71 (9.60)	38.00 (21.00–54.00)	79.49 (8.80)	80.40 (65.05–97.80)
**1021**	14	41.00 (10.02)	43.50 (23.00–54.00)	84.78 (11.20)	84.25 (64.80–102.00)
**1022**	32	35.97 (10.27)	37.00 (20.00–54.00)	82.02 (10.20)	79.83 (66.10–103.00)
**1039**	14	39.29 (10.11)	40.00 (21.00–52.00)	79.93 (8.81)	78.00 (67.00–96.00)
**1051**	14	30.29 (6.67)	29.00 (21.00–43.00)	65.15 (6.26)	63.60 (56.20–73.60)
**All Studies**	98	36.53 (9.92)	37.00 (20.00–54.00)	79.09 (10.94)	78.35 (56.20–103.00)

Kg: kilogram; Max: maximum; Min: minimum; SD: standard deviation.

**Table 3:** Parameter Estimates for Dacomitinib Base Structural Models.

Parameter	Estimate (RSE%)				
	Transit	First-Order Absorption		Combined Zero- and First-Order Absorption	
		Without *t*_lag_	With *t*_lag_	Linked	Independent
*CL* (L/h)	29.2 (4.212)	30.5 (3.131)	31.0 (2.748)	26.1 (4.215)	28.4 (4.894)
*V* (L)	131 (10.840)	790 (0.796)	480 (20.833)	2260 (4.093)	1160 (4.052)
*Q* (L/h)	19.9 (16.583)	23.7 (4.641)	27.9 (18.961)	10.4 (8.135)	4.7 (45.106)
*V*ss (L)	2300 (16.783)	2300 (4.33)	2040 (3.250)	6000 (11.667)	6590 (9.347)
MTT (h)	6.91 (5.731)				
*k_a_* (h^−1^)	0.0246 (12.48)	0.0419 (4.893)	0.0369 (23.55)		0.00966 (9.979)
F	1 FIX	1 FIX	1 FIX	1 FIX	1 FIX
*t*_lag_ (h)			0.749 (8.278)		
D2 (h)				9.78 (2.945)	10.4 (2.481)
* F1				0.455(2.077)	0.0782 (15.729)
Residual error	0.331 (6.133)	0.403 (3.772)	0.346 (5.925)	0.378 (4.259)	0.362 (4.751)

* F1: Fraction absorbed by first order, F1 = 1 − F2.

**Table 4:** Dacomitinib Final Model Pharmacokinetic Parameters Summary.

Parameter	Parameter Estimate	RSE (%)	IIV CV (%)	Shrinkage (%)
Clearance (*CL*, L/h)	29.893	3.03	18.475	21.56
Volume (*V*, L)	789.748	9.47	20.000	63.13
Inter-compartmental Clearance (*Q*, L/h)	76.796	7.62	15.811	77.94
Volume of Distribution at steady-state (*V*ss, L)	2276.46	3.48	15.811	51.68
Mean Transit Time (MTT, h)	12.033	6.00	15.811	70.55
Absorption Rate constant (*k_a_*, h^−1^)	0.259	16.56	54.904	17.09
PPI on *k_a_*	−0.524	−28.34	NA	NA
PPI on *F*	−0.487	−15.10	NA	NA
Relative Bioavailability (*F*)	1 (FIX)	-	23.275	17.17
Proportional Residual Error	0.289	5.23	NA	6.44

CV: coefficient of variation; h: hour; L: liter; NA: not applicable; RSE: relative standard error; IIV: inter-individual variability.

## Supplementary Materials

The following are available online at https://www.mdpi.com/1999-4923/12/4/330/s1, Figure S1: Assignment of pKa to the Dacomitinib Structure, Table S1: Dacomitinib Absorption Models: Base Structural Model Objective Function Value and Condition Number.
