# Supplementary material for: Pharmacokinetic Models to Characterize the Absorption Phase and the Influence of a Proton Pump Inhibitor on the Overall Exposure of Dacomitinib
Source: Pharmaceutics. 2020 Apr 7;12(4):330. doi: 10.3390/pharmaceutics12040330 (PMC7238139; doi:10.3390/pharmaceutics12040330)
Supplement: Supplementary file 1 [file pharmaceutics-12-00330-s001.pdf]

pharmaceutics

Supplementary Materials: Pharmacokinetic Models to Characterize the Absorption Phase and the Influence of a Proton Pump Inhibitor on the Overall Exposure of Dacomitinib

Ana Ruiz-Garcia, Weiwei Tan, Jerry Li, May Haughey, Joanna Masters, Jennifer Hibma, Swan Lin

Table S1. Dacomitinib Absorption Models: Base Structural Model Objective Function Value and Condition Number.

Model	df	Objective Function Value	Condition Number
First-order absorption, without Lag Time	4	−1223.307	1108.33
First-order absorption, with Lag Time	5	−1596.383	944.88
Transit Compartment	5	−1889.846	195.61
Order-zero and first-order absorption Model	6	−1607.126	231.68
Order-zero and first-order linked absorption Model	5	−1480.944	199.57

df: degrees of freedom calculated as number of parameters-1.

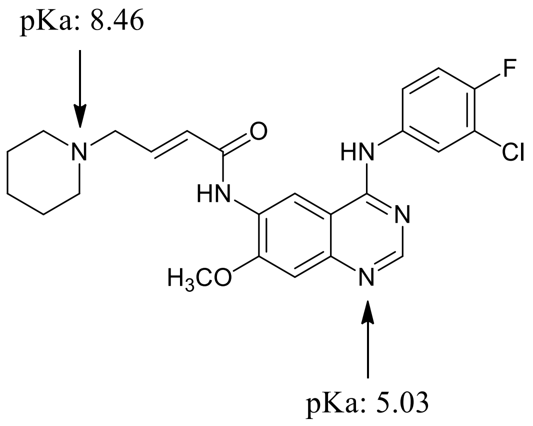

Figure S1. Assignment of pKa to the Dacomitinib Structure.
